# A nationwide survey on clinical practice patterns and bleeding complications of percutaneous native kidney biopsy in Japan

**DOI:** 10.1007/s10157-020-01869-w

**Published:** 2020-03-18

**Authors:** Takehiko Kawaguchi, Tasuku Nagasawsa, Kazuhiko Tsuruya, Kenichiro Miura, Takayuki Katsuno, Takashi Morikawa, Eiji Ishikawa, Masao Ogura, Hideki Matsumura, Ryota Kurayama, Shinsuke Matsumoto, Yuhji Marui, Shigeo Hara, Shoichi Maruyama, Ichiei Narita, Hirokazu Okada, Yoshifumi Ubara, Committee of Practical Guide for Kidney Biopsy 2019

**Affiliations:** 1 Department of Nephrology, National Hospital Organization Chibahigashi National Hospital, 673 Nitona-cho, Chuo-ku, Chiba, 260-8712 Japan; 2 Division of Nephrology, Endocrinology, and Vascular Medicine, Tohoku University Hospital, Sendai, Japan; 3 Department of Nephrology, Nara Medical University, Nara, Japan; 4 Department of Pediatric Nephrology, Tokyo Women’s Medical University, Tokyo, Japan; 5 Department of Nephrology and Rheumatology, Aichi Medical University, Aichi, Japan; 6 Department of Nephrology and Hypertension, Osaka City General Hospital, Osaka, Japan; 7 Department of Nephrology, Mie University, Mie, Japan; 8 Department of Nephrology and Rheumatology, National Center for Child Health and Development, Tokyo, Japan; 9 Department of Pediatrics, Osaka Medical College, Osaka, Japan; 10 Department of Pediatrics, Kosei General Hospital, Tokyo, Japan; 11 Department of Pediatrics, Matsudo City General Hospital, Chiba, Japan; 12 Department of Urology, St. Marianna University School of Medicine, Kawasaki, Japan; 13 Department of Pathology, Kobe City Medical Center General Hospital, Kobe, Japan; 14 Department of Nephrology, Nagoya University, Nagoya, Japan; 15 Division of Clinical Nephrology and Rheumatology, Niigata University, Niigata, Japan; 16 Department of Nephrology, Saitama Medical University, Saitama, Japan; 17 Department of Nephrology, Toranomon Hospital, Tokyo, Japan

**Keywords:** Kidney biopsy, Clinical practice pattern, Bleeding complication, Macroscopic hematuria, Erythrocyte transfusion, Transcatheter arterial embolization

## Abstract

**Background:**

Practice patterns and bleeding complications of percutaneous native kidney biopsy (PNKB) have not recently been investigated and the Japanese Society of Nephrology performed a nationwide questionnaire survey in 2018.

**Methods:**

The survey consisted of nine sections about PNKB: (1) general indications; (2) indications for high-risk patients; (3) informed consent; (4) pre-biopsy evaluation; (5) procedures; (6) sedation; (7) post-biopsy hemostasis, bed rest, and examinations; (8) bleeding complications; and (9) specimen processing. A supplementary survey examined bleeding requiring transcatheter arterial embolization (TAE).

**Results:**

Overall, 220 directors of facilities (nephrology facility [NF], 168; pediatric nephrology facility [PF], 52) completed the survey. Indications, procedures, and monitoring protocols varied across facilities. Median lengths of hospital stay were 5 days in NFs and 6 days in PFs. Gauge 14, 16, 18 needles were used in 5%, 56%, 33% in NFs and 0%, 63%, 64% in PFs. Mean limits of needle passes were 5 in NFs and 4 in PFs. The bed rest period was 16–24 h in 60% of NFs and 65% of PFs. Based on 17,342 PNKBs, incidence rates of macroscopic hematuria, erythrocyte transfusion, and TAE were 3.1% (NF, 2.8%; PF, 6.2%), 0.7% (NF, 0.8%; PF, 0%), and 0.2% (NF, 0.2%; PF, 0.06%), respectively. Forty-six percent of facilities processed specimens all for light microscopy, immunofluorescence, and electron microscopy, and 21% processed for light microscopy only. Timing of bleeding requiring TAE varied among PNKB cases.

**Conclusion:**

Wide variations in practice patterns of PNKB existed among facilities, while PNKBs were performed as safely as previously reported.

**Electronic supplementary material:**

The online version of this article (10.1007/s10157-020-01869-w) contains supplementary material, which is available to authorized users.

## Introduction

Kidney biopsy (KB) is an essential tool for the diagnosis and treatment of patients with numerous kidney diseases. However, its invasive nature results in bleeding complications, leading to increased patient morbidity, longer hospital stay, and higher medical costs. Advancements have been made in biopsy techniques to improve diagnostic yield and to minimize complications, but there remain no global guidelines available to the nephrology community on indications and procedures of KB to improve diagnosis and prognosis.

The Japanese Society of Nephrology (JSN) originally published the “Guidebook of the renal biopsy” in 2004 to develop best strategies for KB and to minimize the risk of complications [1]. Over 10 years have passed and the annual number of percutaneous native kidney biopsies (PNKBs) has recently exceeded 10,000 [2, 3]. The techniques and devices for PNKB have evolved to improve safety profiles, while high-risk patients, such as diabetic and elderly kidney disease patients, have been rapidly increasing in number. However, the clinical practice patterns and bleeding complications of PNKB have never been comprehensively investigated since the publication of the guidebook.

This study aimed to obtain information on the current clinical practice patterns and bleeding complications of PNKB in Japan. The survey could be helpful in providing a reliable consensus and developing better guidelines for PNKB.

## Materials and methods

### Data source

The JSN designed a nationwide questionnaire survey of medical directors of nephrology and pediatric nephrology in Japanese hospitals in 2017. The questionnaire was developed through an iterative review process with the Committee members of the Practical Guide for Kidney Biopsy 2019 of the JSN, to clarify clinical practice patterns and bleeding complications of KB in Japan.

The survey consisted of 70 questions about PNKB, with the exception of transplanted graft biopsy, in nine sections: (1) general indications; (2) indications for high-risk patients; (3) informed consent; (4) pre-biopsy evaluation; (5) biopsy procedures; (6) sedation; (7) post-biopsy hemostasis, bed rest, and examinations; (8) incidence rate of bleeding complications; and (9) specimen processing. The complete survey is described in the supplementary material (Supplement 1).

The JSN distributed the anonymous online survey to nephology teaching hospitals designated by the JSN in February 2018. Respondents were limited to only one director of each facility. Pediatric nephrologists were not asked questions regarding kidney size, since kidney sizes among children vary by age. Conversely, only pediatric nephrologists were asked questions on sedation, since sedation is not routinely performed during PNKB in adult patients. The incidence rates of bleeding complications were investigated over a 3-year period (from January 2015 to December 2017).

The JSN also conducted an anonymous online survey in September 2018 on serious bleeding cases with transcatheter arterial embolization (TAE) after PNKB, which had emerged during the original survey in February 2018. The supplementary questionnaire survey was designed separately from the original to clarify clinical background of hemorrhage events requiring TAE over a period of 5 years (from January 2013 to December 2017). The complete survey is described in the Supplement 2.

These surveys were approved by the ethics committee of the JSN (approvals #53 and #58 in 2018). Multiple approaches to improving participation were implemented, including outreach to the directors through email and verbal contact, reminders through the JSN website, and extending the participation deadline of the surveys.

### Statistical analysis

Standard descriptive statistics were used to describe the practice patterns and incidence rates of complications of PNKB. Percentages, medians (interquartile ranges [IQRs]), and means (standard deviations [SDs]) were reported as appropriate. Data regarding nephrology facilities (NFs) for adult patients and pediatric nephrology facilities (PFs) for child and adolescent patients were analyzed separately, except for the section on specimen processing. In the analyses of incidence rates of bleeding complications, facilities with missing data on minor or major complications were excluded. Only facilities having complete data for both minor and major complications over the 3 years of the survey period were included in the analyses to adequately compare incidence rates of the complications. All statistical analyses were performed using Stata version 14.2 (Stata Corp., College Station, Texas, USA).

## Results

### Respondent demographics

Overall, 224 of 667 directors of Japanese nephrology teaching hospitals responded and of these 220 directors (NF, 168; PF, 52) completed the survey (33% response rate). The annual number of PNKBs performed varied depending on the facilities (range 0–193) and the number of PNKBs in all facilities totaled 24,238 for the 3-year period (average: 8079 per year).

### Section 1: General indications for kidney biopsy

#### Isolated hematuria

Twenty-six percent and 60% of respondents in NFs and PFs, respectively, did not regard isolated hematuria (IH) as an indication for PNKB. Conversely, patients with IH with macroscopic/gross hematuria, suspected of having IgA nephropathy (IgAN) or hereditary nephritis were considered a specific indication (positive response, 38%, 67%, and 62% of all facilities, respectively). Sixty-two percent of NFs, but only 4% of PFs, recommended a biopsy with IH in the presence of dysmorphic urinary red blood cells of glomerular origin.

#### Isolated proteinuria

Seventy percent of respondents from NFs and 50% in PFs did not regard isolated proteinuria (IP) less than 0.5 g/day as an indication for PNKB, while IP of 0.5 g/day or more in chronic kidney disease (CKD) stage G1, G2, and G3 was considered an indication (Table 1). In advanced CKD G stages, the percent of positive responses increased according to proteinuria or higher CKD A stage in NFs, but remained unchanged according to proteinuria in PFs. IP with suspected hereditary nephritis was considered a specific indication (79% of all facilities). Ninety percent of respondents in NFs and 38% in PFs recommended or experienced a biopsy for IP in the presence of dysproteinemia or tubular proteinuria.

**Table 1:** Indications or experiences of kidney biopsy for patients with isolated proteinuria

	Urinary protein							
CKD G stage	≥ 0.15 g/day (%)		≥ 0.5 g/day (%)		≥ 1 g/day (%)		≥ 3.5 g/day (%)	
	NF	PF	NF	PF	NF	PF	NF	PF
G1 (eGFR > 90)	24	29	83	88	98	94	98	94
G2 (eGFR 60–90)	29	44	87	85	98	87	98	87
G3 (eGFR 30–60)	29	52	79	77	96	79	98	81
G4 (eGFR 15–30)	17	31	38	46	52	50	66	52
G5 (eGFR < 15)	8	19	11	27	18	31	31	31

*NF* nephrology facilities for adult patients, *PF* pediatric nephrology facilities for child and adolescent patients

#### Proteinuria with hematuria

In contrast to IP, proteinuria less than 0.5 g/day with hematuria was regarded as an indication for PNKB in over 60% of respondents in NFs and 50% in PFs (Table 2). Hematuria and proteinuria of 0.5 g/day or more in CKD stage G1, G2, and G3 were considered an indication. In advanced CKD G stages, the proportion of positive answers increased according to proteinuria or higher CKD A stage in NFs, but remained unchanged according to proteinuria status in PFs.

**Table 2:** Indications or experiences with kidney biopsy for patients with concomitant proteinuria and hematuria

	Urinary protein							
CKD G stage	≥ 0.15 g/day (%)		≥ 0.5 g/day (%)		≥ 1 g/day (%)		≥ 3.5 g/day (%)	
	NF	PF	NF	PF	NF	PF	NF	PF
G1 (eGFR > 90)	64	52	96	100	99	100	99	100
G2 (eGFR 60–90)	67	65	98	92	100	92	100	92
G3 (eGFR 30–60)	61	62	91	79	97	79	98	79
G4 (eGFR 15–30)	32	38	48	48	58	50	68	52
G5 (eGFR < 15)	15	25	25	31	30	31	39	33

*NF* nephrology facilities for adult patients, *PF* pediatric nephrology facilities for child and adolescent patients

#### Rapidly progressive glomerulonephritis or acute renal failure

PNKB for rapidly progressive glomerulonephritis (RPGN), such as antineutrophil cytoplasmic antibody (ANCA)-associated nephritis and anti-glomerular basement membrane (GBM) nephritis, and acute renal failure (ARF), including drug-induced nephropathy, were recommended or performed in most facilities (positive response for RPGN: 98% of NFs and 89% of PFs; for ARF: 89% of NFs and 67% of PFs). However, the indication for PNKB for RPGN or ARF varied according to kidney function. The proportion of positive responses for RPGN or ARF with estimated glomerular filtration rate (eGFR) less than 60, 30, and 15 mL/min/1.73 m^2^ was 89%, 79%, and 63% of NFs, and 71%, 50%, and 33% of PFs, respectively.

#### Systemic diseases

High proportions of all respondents gave positive answers for PNKB indications involving urinary abnormalities with systemic diseases, such as systemic lupus erythematosus (SLE, 96%) and vasculitis (VA, 97%). Interestingly, SLE and VA with no urinary abnormalities also presented indications for PNKB in a number of facilities (SLE: 45% of NFs and 81% of PFs, VA: 52% of NFs and 29% of PFs). Other diseases or conditions not associated with urinary abnormalities were also indications for PNKB, including enlarged kidney or kidney dysfunction of unknown cause; IgG4-related diseases; hypocomplementemia; hereditary diseases, such as Fabry disease; and positive tubular injury markers, such as β2 microglobulin (MG), α1 MG, and *N*-acetyl-β-d glucosaminidase.

#### Diabetic kidney disease

Diabetes mellitus (DM) with urinary abnormalities was an indication for PNKB in 88% of respondents in NFs and 11% in PFs. Surprisingly, only 2% of NFs, but 38% of PFs had no experience with PNKB in cases with diabetic kidney disease (DKD). Indications for PNKB of DKD varied according to CKD G stage and proteinuria (Table 3). DKD with hematuria, rapid increase in proteinuria, and absence of retinopathy were also considered an indication for PNKB in NFs (88%, 88%, and 89%, respectively).

**Table 3:** Indications or experiences of kidney biopsy for patients with diabetic kidney disease

	Urinary protein									
CKD G stage	< 0.15 g/day (%)		≥ 0.15 g/day (%)		≥ 0.5 g/day (%)		≥ 1 g/day (%)		≥ 3.5 g/day (%)	
	NF	PF	NF	PF	NF	PF	NF	PF	NF	PF
G1 (eGFR > 90)	10	2	19	10	42	13	64	17	77	63
G2 (eGFR 60–90)	12	4	21	6	45	10	68	12	82	12
G3 (eGFR 30–60)	14	4	22	8	43	10	64	12	75	12
G4 (eGFR 15–30)	8	4	11	6	23	6	30	6	36	6
G5 (eGFR < 15)	4	2	4	2	7	2	10	2	14	2

*NF* nephrology facilities for adult patients, *PF* pediatric nephrology facilities for child and adolescent patients

#### Elderly patients

The upper age limit for PNKB was 79 years in 20%, 89 years in 57%, and 90 years or older in 13% of NFs, while a minority of nephrologists regarded less than 75 years of age as an indication for PNKB (the upper age limit of 64 years in 0%, 69 years in 2%, and 74 years in 6% of NFs).

#### Hereditary diseases

A number of respondents in both NFs and PFs regarded hereditary diseases as an indication for PNKB or experienced a PNKB of hereditary diseases, such as Alport syndrome (positive answer, 74%), mitochondrial disease (22%), nephronophthisis (27%), and autosomal dominant tubulointerstitial kidney disease (12%).

### Section 2: Indications for kidney biopsy in high-risk patients

Table 4 shows the indications or experience with KB in high-risk patients. High-risk patients were not necessarily contraindicated for PNKB in many facilities of nephrology experts. The survey also indicated that the median of the upper limit of body mass index (BMI) for KB was 35 (IQR 30–35). Additionally, 33% of respondents had experienced a case diagnosed with malignancy by KB in NFs, while there was no experience in PFs.

**Table 4:** Indications or experiences of kidney biopsy for patients at high risk

	Kidney biopsy							
Risks	Echo-guided (%)		Open (%)		Laparoscopic (%)		Total (%)	
	NF	PF	NF	PF	NF	PF	NF	PF
Unilateral kidney or unilateral atrophic or hypoplastic kidney	15	12	20	21	7	0	42	33
Bilateral atrophic or hypoplastic kidney	18	14	3	8	4	0	25	22
Horseshoe kidney	7	8	5	2	1	0	13	10
Cystic kidney disease, including ADPKD, ARPKD, nephronophthisis, and ADTKD	11	31	2	4	0	0	13	35
Hydronephrosis, including retroperitoneal fibrosis and lupus cystitis	12	21	2	2	1	0	15	24
Malignant hypertension, including scleroderma crisis	52	0	1	2	1	0	54	2
Platelet count less than 50,000, including APS, HUS/TTP, TAFRO	23	6	3	8	1	2	27	16
Pregnancy	10	0	1	0	0	0	11	0
Severe obesity	49	19	2	2	2	0	53	21

*NF* nephrology facilities for adult patients, *PF* pediatric nephrology facilities for child and adolescent patients, *ADPKD* autosomal dominant polycystic kidney disease, *ARPKD* autosomal recessive polycystic kidney disease, *ADTKD* autosomal dominant tubulointerstitial kidney disease, *APS* antiphospholipid syndrome, *HUS* hemolytic uremic syndrome, *TTP* thrombotic thrombocytopenic purpura, *TAFRO* thrombocytopenia, anasarca, myelofibrosis, renal dysfunction, and organomegaly

### Section 3: Informed consent for kidney biopsy

PNKB was commonly performed in inpatient settings and only one NF (0.5% overall) performed PNKB in the outpatient setting. Informed consent (IC) was obtained for PNKB in an outpatient consultation room (65% of all facilities), at bedside after hospitalization (55%), or in a patient interview room (31%). IC for blood transfusion was routinely obtained in advance of PNKB only in 26% of NFs and 35% of PFs. Clinical paths were utilized for hospitalization of PNKB in 88% of NFs and 77% of PFs. The lengths of hospital stay were 4–6 days in 79% of NFs and 6 days or more in 71% of PFs. PNKB was performed the next day after admission in 77% of all facilities.

### Section 4: Pre-biopsy evaluations

Pre-biopsy evaluations included assessments of bleeding diathesis, kidney function, kidney size, blood pressure, and anemia. The cutoff values of contraindications for PNKB are described in Table 5. Prothrombin time-international normalized ratio (PT-INR), activated partial thromboplastin time (APTT), bleeding time (BT), and platelet count were screened for indications for PNKB in 83%, 63%, 40%, and 98% of NFs, and 81%, 77%, 31%, and 100% of PFs. Platelet transfusions were performed when platelet counts were below the cutoff value in 51% of NFs and 57% of PFs. Serum creatinine and eGFR were assessed for indication for PNKB in 57% and 51% of NFs, and in 46% and 60% of PFs, respectively. The cutoff values of contraindications for PNKB varied enormously. Major axis and cortical thickness of the kidneys were evaluated to determine indication in 73% and 48% of NFs. Systolic and diastolic blood pressure (BP) were assessed for biopsy indication in 76% and 39% of NFs, and in 42% and 29% of PFs, respectively. Hemoglobin level was screened for indication for PNKB in 82% of NFs and 62% of PFs. Erythrocyte transfusion (ET) was performed below the cutoff value in 63% of NFs and 33% of PFs.

**Table 5:** Cutoff values of contraindications for kidney biopsy

	NF median (IQR), mean (SD)	PF median (IQR), mean (SD)
Bleeding diathesis		
PT-INR	1.5 (1.5–1.6), 1.6 (0.3)	1.5 (1.5–2.0), 1.6 (0.4)
APTT (s)	45 (40–50), 47 (11)	50 (45–50), 52 (16)
Bleeding time (min)	5.0 (5.0–5.0), 4.8 (1.6)	5 (5–5), 4.8 (0.6)
Platelet count, × 10^4^/mm^3^	5.0 (5.0–10.0), 6.6 (2.5)	9.0 (5.0–10.0), 7.5 (3.2)
Kidney function		
Serum creatinine, mg/dl	3.0 (2.0–3.0), 3.2 (1.5)	3.3 (3.0–4.0), 4.0 (2.3)
eGFR, ml/min/1.73 m^2^	30 (15–30), 25 (9.2)	30 (20–30), 29 (12)
Kidney size^a^		
Major axis, mm	80 (80–90), 83 (68)	-
Cortical thickness, mm	9 (5–10), 8.8 (4.5)	-
Blood pressure		
Systolic blood pressure, mmHg	180 (160–180), 175 (18)	150 (140–155), 153 (20)
Diastolic blood pressure, mmHg	100 (100–110), 106 (9)	100 (90–100), 96 (7)
Anemia		
Hemoglobin, g/dL	8.0 (7.0–9.0), 8.0 (1.1)	7.0 (6.0–8.0), 7.1 (1.2)

*NF*, nephrology facilities for adult patients, *PF* pediatric nephrology facilities for child and adolescent patients

^a^Pediatric nephrologists were not asked the questions on kidney size, because kidney sizes of children vary depending on age

### Section 5: Procedures of kidney biopsy

#### Sampling of specimens

KB was usually performed percutaneously under ultrasonic guidance with local anesthesia in all facilities, but open biopsy (17% of NFs and 56% of PFs) and laparoscopic biopsy (5% of NFs and 0% of PFs) were performed for high-risk patients. Automatic biopsy needles (biopsy gun) were utilized for KB in 98% of NFs and 96% of PFs, while Tru-Cut needles and Silverman needles were used in only a few facilities (0.9% and 1.4% of all facilities). Gauge 14, 16, and 18 needles were usually used for KB in 5%, 56%, and 33% of NFs and in 0%, 63%, and 37% of PFs, respectively. The maximum number of sampled specimens for KB varied across facilities. Two specimens were sampled in 17% of NFs and 56% of PFs and three specimens in 38% of NFs and 23% of PFs. There was also variation in the limit of needle passes for sampling specimens. Three, 4, and 5 passes at a maximum were allowed in 11%, 15%, and 26% of NFs, and 17%, 25%, and 17% of PFs, respectively. Thirty-three percent of NFs and 27% of PFs had no limit of needle passes for sampling specimens.

#### Maximal barrier precautions

Maximal barrier precautions for KB included the use of a cap (positive response, 85% of NFs and 79% of PFs), a mask (97% of all), sterile body gown (68% of PFs and 54% of PFs), sterile gloves (98% of all), and sterile drape (67% of NFs and 60% of PFs). The proportion of facilities using all equipment and instruments described above was 38% of NFs and 35% of PFs.

#### Medications and urethral catheter use

Antibiotics and atropine were regularly used before PNKB in 62% and 21% of NFs, and 58% and 15% of PFs. An indwelling urethral catheter was routinely used in 56% of NFs and 15% of PFs.

#### Blood pressure and antihypertensive drugs

Sixty-five percent of NFs and 39% of PFs had a target for BP during the PNKB. The target varied among facilities, but the most common target for systolic BP was less than 160 mmHg in NFs (62%) and less than 130 or 140 mmHg in PFs (53%). The most common target for diastolic BP was less than 100 mmHg in both in NFs (49%) and PFs (62%). For adult patients with high BP during KB, antihypertensive agents were administered orally in 28%, sublingually in 4%, and intravenously in 50%, and were not used in 27% of NFs. For pediatric patients, antihypertensive drugs were administered orally in 12%, sublingually in 6%, intravenously in 25%, and were not used in 61% of NFs.

### Section 6: Sedation

Intravenous anesthesia (IVA) in a hospital ward was performed during the PNKB in 78% of PFs and the age of indication for IVA varied vastly across facilities. IVA was indicated for all pediatric cases, or patients aged 15 years or younger, in 42% of facilities. Hydroxyzine was used for induction in 49%. For intravenous anesthetics, midazolam, pentazocine, ketamine, and thiopental or thiamylal were administered in 59%, 56%, 49%, and 34% of PFs, respectively. A wide variation was also found in the age of indication for general anesthesia (GA) in the operating room setting during the PNKB. GA was indicated for patients younger than 3 years in 81% and was indicated for all pediatric cases, or patients aged 15 years or younger, in 12% of PFs. Open biopsy was indicated for patients aged less than 1 year in 86% of PFs.

### Section 7: Post-biopsy hemostasis, bed rest, and examination

#### Hemostasis

Manual compression on the biopsy site was performed soon after PNKB in 95% of NFs and 100% of PFs. The median manual compression periods were 10 (IQR 10–15) min in NFs and 15 (IQR 10–15) min in PFs. Sandbags were used for compression on the biopsy site in 62% of NFs and 63% of PFs. The median compression periods using sandbags were 4 (IQR 2–6) h in NFs and 6 (IQR 3–12) h in PFs. Abdominal taping or bandage was used over the biopsy sites in 78% of NFs and 85% of PFs. The median duration of abdominal taping or bandaging was 16 (IQR 6–20) h in NFs and 20 (IQR 14–24) h in PFs. It depended largely on facilities when discontinued anticoagulant or antiplatelet drugs before PNKB could be restarted after biopsy. The mean duration of drug discontinuation was 2 (IQR 1–7) days in NFs and 7 (IQR 2–7) days in PFs. Hemostatic agents were routinely used after PNKB in 66% of NFs and 60% of PFs. Carbazochrome sodium sulfonate hydrate and tranexamic acid were regularly used in 96% and 100% of NFs and in 83% and 63% of PFs, respectively.

#### Bed rest

The prescribed period of strict bed rest in a supine position after PNKB varied among facilities. Strict bed rest for 4–8 h was prescribed in 44% of NFs and in 33% of PFs. Total bed rest for 16–24 h overall was prescribed in 60% of NFs and in 65% of PFs. The median period of subsequent exercise restriction was 14 (IQR 7–28) days in NFs and 14 (IQR 14–28) days in PFs.

#### Medical examinations and interventions

Blood tests were routinely performed for monitoring patients after the PNKB in 95% of NFs and in 84% of PFs. The examinations were conducted the day after the PNKB in 68% of NFs and 51% of PFs. Ultrasound examinations were routinely performed to confirm hemostasis after KB in 84% of NFs and 82% of PFs and were performed immediately after the KB in 70% of the NFs and in 60% of the PFs. When severe bleeding occurred after the PNKB, surgical procedures could be adopted in their own hospitals in 84% of NFs and 80% of PFs. Transcatheter arterial embolization could also be adopted in their own hospitals in 66% of NFs and 39% of PFs.

### Section 8: Bleeding complications

One hundred and eighty-one of the 220 facilities (138 of 168 NFs and 43 of 52 PFs; 82% of all facilities) and 17,342 of 24,238 PNKBs (15,657 of 21,468 from NFs and 1685 of 2770 from PFs: 72% of all PNKBs) were included in the analyses of incidence rates of bleeding complications. Tables 6, 7 and 8 show the incidence of minor and major bleeding complications after PNKB procedures in Japanese facilities. A minor bleeding complication was defined as a macroscopic hematuria (MH) with no treatment except for rehydration, and major bleeding complications included need for blood transfusion, surgical hemostasis, TAE, bladder lavage and/or nephrectomy and death from severe bleeding. Based on 17,342 PNKBs performed (15,657 in NFs and 1685 in PFs) over the past 3 years, the overall incidence rates of MH, ET, and TAE were 3.1% (95% confidence interval [CI] 2.8–3.4), 0.7% (95% CI 0.6–0.8), and 0.2% (95% CI 0.1–0.3), respectively (Table 6). The incidence rates of MH, ET, and TAE in NFs were 2.8% (95% CI 2.5–3.0), 0.8% (95% CI 0.6–0.9), and 0.2% (95% CI 0.1–0.3) and in PFs were 6.2% (95% CI 5.1–7.5), 0%, and 0.06% (95% CI 0.002–0.3) (Tables 7, 8). Death was attributed to severe bleeding in only one adult case of all PNKBs performed [0.006% (95% CI 0.0001–0.03)].

**Table 6:** Bleeding complications of percutaneous native kidney biopsies in all facilities of nephrology and pediatric nephrology

Year	2015	2016	2017	Total
Number of facilities	147	146	154	447
Number of biopsies	5548	5691	6103	17,342
Macroscopic hematuria with no treatment (%)	160 (2.9)	178 (3.1)	198 (3.3)	536 (3.1)
Bleeding complications with treatment (%)	60 (1.1)	54 (1.0)	64 (1.0)	178 (1.0)
Erythrocyte transfusion^a^ (%)	38 (0.7)	39 (0.7)	44 (0.7)	121 (0.7)
Surgical hemostasis^a^ (%)	1 (0.02)	0 (0)	0 (0.0)	1 (0.006)
Transcatheter arterial embolization^a^ (%)	14 (0.3)	8 (0.1)	10 (0.2)	32 (0.2)
Bladder lavage^a^ (%)	23 (0.4)	17 (0.3)	25 (0.4)	65 (0.4)
Nephrectomy^a^ (%)	0 (0)	0 (0)	0 (0)	0 (0)
Death from severe bleeding^a^ (%)	0 (0)	0 (0)	1 (0.02)	1 (0.006)

^a^Multiple selections allowed in the same biopsy

**Table 7:** Bleeding complications of percutaneous native kidney biopsies in nephrology facilities for adult patients

Year	2015	2016	2017	Total
Number of facilities	112	114	117	343
Number of biopsies	4906	5192	5559	15,657
Macroscopic hematuria with no treatment (%)	132 (2.7)	136 (2.6)	163 (2.9)	431 (2.8)
Bleeding complications with treatment (%)	58 (1.2)	50 (1.0)	59 (1.1)	167 (1.1)
Erythrocyte transfusion^a^ (%)	38 (0.8)	39 (0.8)	44 (0.8)	121 (0.8)
Surgical hemostasis^a^ (%)	1 (0.02)	0 (0)	0 (0.0)	1 (0.006)
Transcatheter arterial embolization^a^ (%)	14 (0.3)	8 (0.2)	9 (0.2)	31 (0.2)
Bladder lavage^a^ (%)	21 (0.4)	15 (0.3)	20 (0.4)	56 (0.4)
Nephrectomy^a^ (%)	0 (0)	0 (0)	0 (0)	0 (0)
Death from severe bleeding^a^ (%)	0 (0)	0 (0)	1 (0.02)	1 (0.006)

^a^Multiple selections allowed in the same biopsy

**Table 8:** Bleeding complications of percutaneous native kidney biopsies in pediatric nephrology facilities for child and adolescent patients

Year	2015	2016	2017	Total
Number of facilities	35	32	37	104
Number of biopsies	642	499	544	1685
Macroscopic hematuria with no treatment (%)	28 (4.4)	42 (8.4)	35 (6.4)	105 (6.2)
Bleeding complications with treatment (%)	2 (0.3)	4 (0.8)	5 (0.9)	11 (0.7)
Erythrocyte transfusion^a^ (%)	0 (0)	0 (0)	0 (0)	0 (0)
Surgical hemostasis^a^ (%)	0 (0)	0 (0)	0 (0)	0 (0)
Transcatheter arterial embolization^a^ (%)	0 (0)	0 (0)	1 (0.2)	1 (0.06)
Bladder lavage^a^ (%)	2 (0.3)	2 (0.4)	5 (0.9)	9 (0.5)
Nephrectomy^a^ (%)	0 (0)	0 (0)	0 (0)	0 (0)
Death from severe bleeding^a^ (%)	0 (0)	0 (0)	0 (0)	0 (0)

^a^Multiple selections allowed in the same biopsy

### Section 9: Processing of biopsy specimens

Biopsy specimens were fixed and processed in each facility’s laboratory for light microscopy (LM), immunofluorescence (IF), and electron microscopy (EM), in 98%, 78%, and 46% of facilities. Forty-six percent fixed and processed specimens comprehensively for all LM, IF, and EM, and 21% only for LM.

Assistant operators (39%) or biopsy operators (31%) mainly divided biopsy specimens and put them in the fixatives. The specimens were routinely assessed with a microscope before dividing in 60% of facilities (34% with a dissecting microscope and 26% with a light microscope), but were not assessed in 31%. Biopsy specimens were kept from drying before fixation in 84% of facilities. The specimens were wrapped with saline-soaked gauze in 52% and placed directly in normal saline in 24% of the facilities. Each facility determined how biopsy specimens were to be divided for LM, IF, and EM. Either end of the specimen core was taken for IF and EM, with the remainder divided for LM in 40% of facilities, and both ends from all cores were taken for IF and EM, with the remainder divided for LM in 21%. Samples were randomly taken from all cores for IF, EM, and LM in 39% of facilities.

The fixative used was 10% formalin in 61% of facilities, while neutral buffered formalin was used in 22%. Formalin alone was used as a fixative in 68% of the facilities, while both formalin and another fixative, such as picric acid, were used in 16%. Liquid nitrogen, dry ice, dry ice with acetone, hexane bottle in dry ice with acetone, and a deep freezer were used for freezing specimens for processing in 24%, 23%, 17%, 12%, and 9% of biopsy samples, respectively.

### Supplementary survey: Clinical background of bleeding events requiring TAEs after a PNKB

In the supplementary questionnaire survey, 40 patients from 29 NFs were identified who underwent TAEs after PNKB over the previous 5 years. No patients requiring TAEs were reported from PFs.

The median age was 56 (IQR 39–69) years and 53% of the patients were male. The median BMI was 22 (IQR 19–25) and 89% of the patients carried out normal activities of daily living (ADL) without being bedridden or confined to a wheelchair. Approximately 20% had DM and 63% had no high-risk background, such as unilateral/hypoplastic/atrophic kidney, cystic kidney diseases, malignant hypertension, or pregnancy. The PNKBs were all performed in inpatient settings, where anticoagulant and antiplatelet agents were used at biopsy in 7.5% and 5.0% of the cases, respectively. More common among the patients with TAE were chronic glomerulonephritis (31%) and RPGN (28%) in the clinical diagnoses and IgAN (18%) and crescentic glomerulonephritis (15%) in the histological diagnosis. Laboratory values before the PNKB (median [IQR]) were as follows: eGFR, 35 (10–70) mL/min/1.73 m^2^; hemoglobin, 11.0 (9.6–13.2) g/dL; platelet count, 21.5 (15.5–29.9) × 10^4^/μL; PT-INR, 1.0 (1.0–1.2); APTT, 29 (27–33) s; and BT, 2.5 (2.0–4.0) min. Mean systolic and diastolic BP at the PNKB were 137 (IQR 119–158) and 80 (IQR 72–90) mmHg, respectively. Median kidney size (major axis) was 100 (IQR 96–109) mm.

Table 9 shows the procedure used for the PNKB as well as the timing and type of hemorrhage occurring after the PNKB, for those patients who underwent TAE. The biopsy procedures, such as the number of needle passes for sampling specimens and the period of bed rest, varied among the cases. The PNKBs resulted in peri-nephric or intra-nephric bleeding in 87% of the cases, while only macrohematuria without peri-nephric or intra-nephric bleeding occurred in 13%. Bleeding requiring TAE was apparent within 6 h after the PNKB in 53% and within 24 h in 65% of the cases, while it became evident after 1 week or later in 20%.

**Table 9:** Biopsy procedures and timing of bleeding after biopsy for patients undergoing transcatheter arterial embolization (TAE)

	(%)
Biopsy procedures	
Needle gauge	
14	8
15	62
16	3
17	27
Needle passes for sampling specimens	
1	3
2	32
3	35
4	14
≥ 5	16
Use of hemostatic agents	
Yes	57
No	43
Manual compression on the biopsy site after biopsy	
< 15 min	61
15–30 min	31
≥ 30 min	8
Strict bed rest in a supine position after biopsy	
< 2 h	26
2–4 h	28
4–8 h	30
8–12 h	13
≥ 12 h	3
Total bed rest after biopsy	
< 8 h	13
8–16 h	30
16–24 h	48
≥ 24 h	8
Number of kidney biopsies the operators had experienced	
< 50	32
Before the biopsy for the patient with TAE	
50–200	39
≥ 200	29
Timing of bleeding requiring TAE after biopsy	
Cumulative timing of bleeding	
< 6 h	53
< 12 h	60
< 24 h	65
< 7 days	80
≥ 7 days	20

## Discussion

This nationwide survey describes the clinical practice patterns and bleeding complications of PNKB in Japanese nephrology facilities. This is the first comprehensive report showing the wide variations in clinical practice across facilities and the low rate of bleeding complications observed in Japan when PNKBs are performed using current techniques and devices.

First, the survey extensively investigated indications for PNKB. Previous studies did not recommend performing a biopsy for patients with IH, since many patients with IH might manifest no abnormality or slight changes in histology, requiring no specific therapy with excellent prognosis [4, 5]. The present survey confirmed these data, but the presence of macroscopic/gross hematuria or asymptomatic hematuria of suspected glomerular origin appeared to be a specific indication for biopsy. Under these conditions, biopsy results could alter patient management. One of the most frequent causes of IH is IgAN. IgAN patients with permanent IH may not have a favorable outcome and are at high risk of progression of renal damage [6, 7]. It might be better to diagnose and manage IgAN early to reduce the probability of end-stage kidney disease. The survey also suggested that IP of less than 0.5 g/day was not an indication for PNKB in most cases because of a better prognosis of mild proteinuria without systemic diseases, such as SLE and VA. Conversely, IP in the presence of dysproteinemia or tubular proteinuria or IP of suspected hereditary nephritis was considered a specific indication for biopsy, which could determine specific treatment policy. Concurrent proteinuria and hematuria encouraged nephrologists to perform PNKBs, because the condition could suggest a progressive glomerular disease and predict a poor renal outcome.

RPGN, ARF, and systematic diseases such as SLE and VA presented good indications for PNKB. Interestingly, systemic diseases without urinary abnormalities also presented an indication for PNKB for many nephrologists, since active pathological lesions are often found in patients with systemic diseases without urinary abnormalities (known as “silent lupus nephritis”) [8, 9].

Policies on performing PNKB for patients with DM and elderly patients have been changing. DM is now the leading cause of end-stage kidney disease in the world, and the previously established opinion among nephrologists was that KB for DKD would provide little diagnostic, prognostic, or therapeutic value [10]. However, DKD has been recognized as a new disease entity and a worldwide public health problem [11], and it was revealed in the survey that DM with urinary abnormalities presented an indication for PNKB in many facilities. A previous study suggested that pathology varied enormously across DKD and also included nondiabetic glomerular diseases, such as membranous nephropathy [12]. These pathological findings could be potentially amenable to therapies that might improve kidney outcome [13]. Additionally, CKD is increasingly common in the elderly, and the survey revealed that performing PNKB in very elderly patients was not always a contraindication in many facilities. PNKB in very elderly patients was not associated with any greater risk of complications than that in younger patients [14], and biopsy could also be a valuable diagnostic tool that provides crucial diagnostic and prognostic information, and changes treatment approach in geriatric patients [15].

High-risk patients may be contraindicated for PNKB. Potential contraindications include unilateral kidney, bilateral atrophic or hypoplastic kidney, horseshoe kidney, multiple bilateral cysts, hydronephrosis, malignant hypertension, uncorrectable bleeding diathesis, pregnancy, and severe obesity. In the previous Japanese Guidebook [1], most of these high-risk conditions were regarded as contraindications for PNKB, while alternative biopsy techniques could be considered. Surprisingly, the survey revealed that many nephrologists do not necessarily regard these high-risk patients as contraindicated for PNKB in the current era when the techniques and devices have evolved to improve safety profiles. It would be equally important to consider which patients would not or would benefit from a biopsy. A laparoscopic or open kidney biopsy can be a good alternative in selected circumstances [16].

The survey found common practices before performing PNKB included assessment of bleeding diathesis, kidney function, kidney size, blood pressure, and anemia, but cutoff values for these parameters contraindicating PNKB and transfusion policies varied enormously across facilities. A previous study reported that bleeding complications were associated with APTT, PT-INR, BT, and platelet count [17]. Additional risk factors for bleeding included hypertension, reduced GFR, anemia, older age, and the use of a 14-gauge biopsy needle [18–22]. However, it may be difficult to determine strict cutoff values for these contraindications for PNKB because of the different patient backgrounds among the previous investigations. The cutoff values should be carefully examined based on individual benefit and risk of patients.

PNKB was most commonly performed by nephrologists in inpatient settings, and clinical paths were utilized for hospitalization in many Japanese facilities. IC for PNKB was obtained in outpatient settings or after admission, while IC for blood transfusion was not routinely obtained in advance of biopsy in most facilities. The length of hospital stay was usually 4–6 days in NFs and 6 days or more in PFs. The duration of hospitalization was likely based on recommendations from the previous Japanese guidebook indicating that patients should be discharged 4–7 days after PNKB to allow better monitoring of bleeding complications after hemostasis [1]. Previous studies have reported worldwide variation in PNKB performed as an inpatient or outpatient procedure, and suggested that the outpatient setting would be safe with low complication rates and more cost-effective than inpatient biopsies [23, 24]. Most bleeding complications are recognized within 12–24 h of the biopsy, but complications occurring after 24 h were not extremely rare (2–10%) [19, 25, 26]. Thus, it might be better to consider the observation period after PNKB based on the patient risk of bleeding complications.

PNKB was most commonly performed with automatic biopsy needles under local anesthesia, while IVA or GA was often performed during pediatric PNKB. Gauge 16 needles were used in many facilities, but a wide variation was found among facilities in the maximum number of sampled specimens and needle passes. A previous systematic review suggested that the use of 14-gauge needles was associated with higher transfusion rates compared with 16- and 18-gauge needles, and that the number of passes did not affect the rate of complications [20]. Additionally, the diagnostic yield did not seem to differ significantly among 14- and 16-gauge needles, while some studies indicate a lower yield with 18-gauge needles [27–30]. These data suggest that the use of 16-gauge needles might be encouraged in most circumstances.

Concerning the prevention of infections, only a minority of facilities used all the equipment and instruments for maximal barrier precautions, while many facilities routinely administered antibiotics regularly for prevention of infections. Although infections during PNKB rarely occurred using sterile techniques, infectious complications have been described in some case series [31], and preventive measures should be carefully taken.

Policies on hemostasis, bed rest, and examination after PNKB varied enormously among facilities. Manual compression on the biopsy site was performed soon after the PNKB in almost all facilities, while it depended on the facilities whether sandbags and abdominal taping/bandage were used for compression. As manual compression and abdominal bandaging may increase the frequency of reflex syncope during PNKB [32], it may be necessary to consider the potential benefit and risk of these maneuvers. Hemostatic agents were routinely administered after PNKB in many facilities, but desmopressin was not used in any of the facilities due to non-coverage by medical insurance in Japan. There was also a wide variation in the prescribed period of bed rest after the PNKB. A shorter period of strict bed rest might safely reduce discomfort in PNKB [33]. Conversely, indwelling urethral catheters were routinely used in many nephrology facilities (but only in a few pediatric facilities) to ensure bed rest and to monitor bleeding. It depended on facilities when a discontinued anticoagulant or antiplatelet drug would be restarted after the PNKB. Not only bleeding risk, but also thrombosis risk, after the PNKB should be considered for patients taking these drugs. Ultrasound examinations, as well as blood tests, were routinely performed to confirm hemostasis after the PNKB in many facilities, although their utility in predicting relevant clinical complications or altering management has not been clearly demonstrated in previous studies [34, 35].

The present survey showed that the incidence rates of MH (3.1%), ET (0.7%), TAE (0.2%), and death attributed to biopsy (0.006%) were as low as those reported in a previous systematic review in 2013 (MH 3.5%, ET 0.9%, TAE 0.6%, death 0.02%) [21] and in a recent Japanese report in 2015 (ET 0.5%, TAE 0.1%, death 0.06%) [3]. It would be critically important to show the safety of PNKB in the modern era of increased number of PNKB performed in high-risk patients, such as elderly patients with many comorbidities. Although our study revealed that the rates of bleeding complications were relatively low when native kidney biopsies were performed using current techniques and devices in Japan, it is crucial to identify which factors of PNKB are significantly associated with the bleeding complications. Further investigations are needed to examine the associations between clinically relevant factors and the complications observed following PNKB procedures in Japan.

In Japanese facilities performing KB, biopsy specimens were not necessarily fixed and processed in their own laboratories all for LM, IF, and EM. It largely depended on facilities how the specimens were to be divided, fixed and processed. Specimens for LM, IF, and EM require different processing and fixation methods, and it is important for nephrologists to be competent at specimen division and processing for optimum diagnostic and prognostic yield [36, 37].

The supplementary survey clarified the clinical backgrounds of severe hemorrhage requiring TAE after PNKB. The original survey did not reveal a high incidence rate of TAE, but TAE was undoubtedly a serious bleeding complication following the PNKB. There have been a few case reports describing TAE after PNKB [38–40], but the underlying conditions for TAE requirement after PNKB have never been comprehensively examined to date. This additional survey suggested a wide variety of patient characteristics and biopsy procedures among cases requiring TAE. Hemorrhage leading to TAE was apparent most frequently within 6 h, while the timing of bleeding with TAE varied widely. Surprisingly, hemorrhage resulting in TAE became evident a week or later after PNKB in 20% of the cases. To the best of our knowledge, this is the first study to report on the timing of bleeding complications associated with TAE requirement. It may be difficult to predict when critical bleeding requiring TAE will be identified, while nearly 90% of all minor and major bleeding complications occur within 24 h after PNKB [20, 25]. This information is crucially important to ensure safety of PNKB. Close patient follow-up is necessary to detect both early and late onset of severe bleeding complications after PNKB.

A strength of our survey is the nationwide investigation providing extensive information on clinical practice patterns and bleeding complications of PNKB in the current era in Japan. The number of PNKBs performed in all the facilities participating in this survey amounted to 24,238 (17,342 alone for analyses of complications) over a 3-year period and was sufficiently large to describe the current approach of the Japanese nephrology community towards PNKB. Despite being only descriptive analyses, the results of the survey are still impressive and informative. The reported practice patterns and estimates of complications provided helpful information in the decision-making process involved for PNKB procedures.

Despite its strengths, the limitations of our survey should be recognized. First, the study was based on a cross-sectional descriptive design, and the reported practice patterns were mostly determined by personal experience and the confidence of the physician with the procedure and were not necessarily based on clinical evidence. It is underlined that the application of the results from this study to clinical practice for PNKB should be done carefully. Next, this was the online questionnaire survey of facility directors, and the response rate was 33% (220 of 667 facilities). The survey presented a lower response rate than the previous questionnaire surveys among nephrology directors (60–80%) [1, 5, 41, 42], which may have led to a potential selection bias. The survey was distributed to Japanese teaching hospitals, to which many patients at high risk might be referred, but facilities with high complication rates of PNKB may less likely have responded to the survey, resulting in an underestimation of complications.

In conclusion, the present survey showed wide variations of clinical practice patterns of PNKB according to facilities, while PNKBs were performed as safely as previously reported in Japan. These findings may help provide a reliable consensus among nephrologists and develop better guidelines for PNKB.

## Electronic supplementary material

Below is the link to the electronic supplementary material.

Supplementary file1 (DOCX 53 kb)

Supplementary file2 (DOCX 27 kb)
