# Supplementary material for: A nationwide survey on clinical practice patterns and bleeding complications of percutaneous native kidney biopsy in Japan
Source: Clin Exp Nephrol. 2020 Mar 18;24(5):389–401. doi: 10.1007/s10157-020-01869-w (PMC7174253; doi:10.1007/s10157-020-01869-w)
Supplement: Supplementary file 1 — Supplementary file1 (DOCX 53 kb) [file 10157_2020_1869_MOESM1_ESM.docx]

Supplement 1

**Questionnaire Form**

**Respondent’s specialty** ① Nephrology ② Pediatrics ③ Urology ④ Other ( Fill in )

**1. General indications for kidney biopsy (KB)**

**(1) When a patient has isolated hematuria, which of the following is indicated for KB, or have you experienced KB with?** (Multiple selections allowed)

① Macroscopic/Gross hematuria ② Red blood cells in the urine sediment (URBCs) >30/hpf

③ URBCs 6-30/hpf ④ Presence of dysmorphic URBCs

⑤ Suspected of having IgA nephropathy ⑥ Suspected of having hereditary nephritis

⑦ Not indicated for KB with isolated hematuria ⑧ Other (Fill in)

**(2) When a patient has isolated proteinuria, which of the following is indicated for KB, or have you experienced KB with?** (Multiple selections allowed)

　　① Indication for KB according to CKD stage (eGFR) and proteinuria

|  | **Urinary protein** | | | |
| --- | --- | --- | --- | --- |
| **CKD G stage** | **≥0.15 g/day** | **≥0.5 g/day** | **≥1 g/day** | **≥3.5 g/day** |
| **G1 (eGFR >90)** | Yes / No | Yes / No | Yes / No | Yes / No |
| **G2 (eGFR 60–90)** | Yes / No | Yes / No | Yes / No | Yes / No |
| **G3 (eGFR 30–60)** | Yes / No | Yes / No | Yes / No | Yes / No |
| **G4 (eGFR 15–30)** | Yes / No | Yes / No | Yes / No | Yes / No |
| **G5 (eGFR <15)** | Yes / No | Yes / No | Yes / No | Yes / No |

② Suspected of hereditary nephritis ③ Dysproteinemia or tubular proteinuria

④ Other ( Fill in )

**(3) When a patient has concomitant proteinuria and hematuria, which of the following is indicated for KB, or have you experienced KB with?** (Multiple selections allowed)

Indication for KB according to CKD stage (eGFR) and proteinuria

|  | **Urinary protein** | | | |
| --- | --- | --- | --- | --- |
| **CKD G stage** | **≥0.15 g/day** | **≥0.5 g/day** | **≥1 g/day** | **≥3.5 g/day** |
| **G1 (eGFR >90)** | Yes / No | Yes / No | Yes / No | Yes / No |
| **G2 (eGFR 60–90)** | Yes / No | Yes / No | Yes / No | Yes / No |
| **G3 (eGFR 30–60)** | Yes / No | Yes / No | Yes / No | Yes / No |
| **G4 (eGFR 15–30)** | Yes / No | Yes / No | Yes / No | Yes / No |
| **G5 (eGFR <15)** | Yes / No | Yes / No | Yes / No | Yes / No |

**(4) Which of the following is indicated for KB, or have you experienced KB with?**

(Multiple selections allowed)

① Rapidly progressive glomerulonephritis ② Acute renal failure (e.g. drug-induced nephropathy)

③ eGFR ≤60 ④ eGFR ≤30 ⑤ eGFR ≤15 ⑥ Other (Fill in)

**(5) When a patient has proteinuria or/and hematuria, which of the following systemic diseases is indicated for KB, or have you experienced KB with?**

(Multiple selections allowed)

　　① Diabetes mellitus ② Systemic lupus erythematosus ③ Vasculitis (e.g. ANCA-associated)

④ Dysproteinemia, such as multiple myeloma and amyloidosis ⑤ Other ( Fill in )

**(6) When a patient has neither proteinuria nor hematuria, which of the following is indicated for KB, or have you experienced KB with?** (Multiple selections allowed)

① CKD stage G2 ② CKD stage G3 ③ CKD stage G4 ④ CKD stage G5

⑤ Systemic lupus erythematosus (“silent” lupus nephritis) ⑥ Vasculitis

⑦ Positive tubular injury markers, such as β2MG, α1MG, and NAG ⑧ Other ( Fill in )

**(7) When a patient has diabetic kidney disease (DKD), which of the following is indicated for KB or have you experienced KB with?** (Multiple selections allowed)

① Indication for KB according to CKD stage (eGFR) and proteinuria

|  | **Urinary protein** | | | | |
| --- | --- | --- | --- | --- | --- |
| **CKD G stage** | **<0.15 g/day** | **≥0.15 g/day** | **≥0.5 g/day** | **≥1 g/day** | **≥3.5 g/day** |
| **G1 (eGFR >90)** | Yes / No | Yes / No | Yes / No | Yes / No | Yes / No |
| **G2 (eGFR 60–90)** | Yes / No | Yes / No | Yes / No | Yes / No | Yes / No |
| **G3 (eGFR 30–60)** | Yes / No | Yes / No | Yes / No | Yes / No | Yes / No |
| **G4 (eGFR 15–30)** | Yes / No | Yes / No | Yes / No | Yes / No | Yes / No |
| **G5 (eGFR <15)** | Yes / No | Yes / No | Yes / No | Yes / No | Yes / No |

② Retinopathy ③ No retinopathy ④ Hematuria ⑤No hematuria ⑥ Rapidly increased proteinuria ⑦ No indication for KB of DKD, or no experience with KB of DKD ⑧ Other ( Fill in )

**(8) Which age is the upper limit to indicate KB, or have you experience with KB in elderly patients?** (Multiple selections allowed)

① <65 years ② <70 years ③ <75 years ④ <80 years ⑤ <90 years ⑥ ≥90 years

⑦ Other ( Fill in ) ⑧No indication for KB in the elderly, or no experience with KB in the elderly

**(9) Which of the following hereditary diseases is an indication for KB, or have you experience with KB for?** (Multiple selections allowed)

① No indication for KB in hereditary nephritis ② Alport syndrome

③ Mitochondrial disease (m.3243 A>G variant) ④ Mitochondrial disease (others)

⑤ Nephronophthisis ⑥ADTKD, including MCKD ⑦ Other ( Fill in )

**2. Indications for kidney biopsy (KB) in patients at high risk**

**Which of the following is an indication for KB, or have you experience with KB for 3 years for?**

**(Multiple selections allowed)**

**(1) Unilateral kidney, or unilateral atrophic or hypoplastic kidney**

**(2) Bilateral atrophic or hypoplastic kidney**

**(3) Horseshoe kidney**

**(4) Cystic kidney disease, including ADPKD, ARPKD, nephronophthisis, and ADTKD, with renal parenchyma confirmed**

**(5) Hydronephrosis, including retroperitoneal fibrosis and lupus cystitis, with renal parenchyma confirmed**

**(6) Malignant hypertension, including scleroderma crisis**

**(7) Platelet count <50,000, including APS, HUS / TTP, TAFRO**

**(8) Pregnancy**

**(9) Severe obesity**

|  | **(1)** | **(2)** | **(3)** | **(4)** | **(5)** | **(6)** | **(7)** | **(8)** | **(9)** |
| --- | --- | --- | --- | --- | --- | --- | --- | --- | --- |
| **Not experience** | Yes  No | Yes  No | Yes  No | Yes  No | Yes  No | Yes  No | Yes  No | Yes  No | Yes  No |
| **Echo-guided biopsy** | Yes  No | Yes  No | Yes  No | Yes  No | Yes  No | Yes  No | Yes  No | Yes  No | Yes  No |
| **Laparoscopic biopsy** | Yes  No | Yes  No | Yes  No | Yes  No | Yes  No | Yes  No | Yes  No | Yes  No | Yes  No |
| **Open biopsy** | Yes  No | Yes  No | Yes  No | Yes  No | Yes  No | Yes  No | Yes  No | Yes  No | Yes  No |

**(10) Have you ever experienced a case diagnosed with malignancy by KB?** (a) Yes (b) No

**(11) Upper limit of the BMI for KB in your facility** ( Fill in )

**(12) Other comments** ( Fill in )

**3. Informed consent for kidney biopsy (KB)**

**(1) Where do you obtain informed consent for KB?**

① In an outpatient consultation room ② At the bedside after hospitalization

③ In a patient interview room ④ Other option ( Fill in )

**(2) Are clinical paths utilized for hospitalization of KB?** ① Yes ② No ③ Other option ( Fill in )

**(3) What is the length of hospital stay for KB?**

　 ① 1 day (no hospitalization) ② 2 days and 1 night ③ 3 days and 2 nights ④ 4 days and 3 nights

　 ⑤ 5 days and 4 nights ⑥ 6 days and 5 nights ⑦ 7 days or more ⑧ Other options ( Fill in )

**(4) When is KB performed during hospitalization?**

① On the day of the outpatient consultation (no hospitalization) ② On the day of admission

③ On the day after admission ④ Other option ( Fill in )

**(5) Do you routinely obtain informed consent for blood transfusion?** ① Yes ② No

**4. Medical evaluations before kidney biopsy (KB)**

**(1) Evaluation of bleeding diathesis; contraindications for KB with bleeding diathesis**

① Is bleeding time evaluated before KB?

(a) Yes. ( Fill in ) seconds or more is a contraindication for KB. (b) No

② Is platelet count evaluated for indication of KB?

(a) Yes. The cutoff value is ( Fill in ) × 10^4^/mm^3^ or fewer. (b) No

③ With the described cutoff or less, is platelet transfusion performed before KB?

(a) Yes (b) No, KB is contraindicated and not performed. (c) Other option ( Fill in )

④ Is PT-INR evaluated before KB? (a) Yes. The cutoff value is ( Fill in ) or more. (b) No.

⑤ Is APTT evaluated before KB? (a) Yes. The cutoff value is ( Fill in ) or more. (b) No.

**(2) Evaluation of kidney function; contraindications for KB with advanced CKD**

① Serum creatinine: (a) Contraindicated for KB with ( Fill in ) mg/dL or more

(b) No limitation (c) Other option ( Fill in )

② eGFR: (a) Contraindicated for KB with ( Fill in ) mL/min/1.73 m^2^ or less

(b) No limitation (c) Other option (Fill in)

**(3) Evaluation of kidney size; contraindications for KB with atrophic kidney**

① Kidney major axis: (a) Contraindicated for KB with ( Fill in ) mm or shorter

(b) No limitation (c) other option ( Fill in )

② Cortical thickness: (a) Contraindicated for KB with ( Fill in ) mm or shorter

(b) No limitation (c) Other option ( Fill in )

**(4) Evaluation of blood pressure; contraindications for KB with high blood pressure**

① Systolic blood pressure: (a) Contraindicated for KB with ( Fill in ) mmHg or higher

(b) No limitation (c) Other option ( Fill in )

② Diastolic blood pressure: (a) Contraindicated for KB with ( Fill in ) mmHg or higher (b) No limitation (c) Other option (Fill in)

**(5) Evaluation of anemia; contraindications for KB with severe anemia**

Is hemoglobin evaluated as an indication of KB?

(a) Yes. The cutoff value is ( Fill in ) g/dL or less. (b) No. (c) Other option ( Fill in )

**(6) With the described cutoff or less, is red blood cell transfusion performed before KB?**

(a) Yes (b) No, KB is contraindicated and is not performed. (c) Other option ( Fill in )

**5. Procedures of kidney biopsy (KB)**

**(1) What procedures do you usually adopt for KB?**

① Ultrasound-guided biopsy ② Open biopsy ③ Laparoscopic biopsy ④ Other option ( Fill in )

**(2) What procedures do you mainly adopt for KB in high-risk patients?**

① Ultrasound-guided biopsy ② Open biopsy ③ Laparoscopic biopsy ④ Other option ( Fill in )

**(3) What type of needles do you usually use for KB?**

① Automatic biopsy needle (biopsy gun) ②Tru-Cut needle ③ Silverman needle

④ Other ( Fill in )

**(4) What size of biopsy needle (gauge (G) x length) do you usually use for KB?**

①14G × 19 mm or smaller ②14G × 20 mm or larger ③16G × 19 mm or smaller

④16G × 20 mm or larger ⑤18G × 19 mm or smaller ⑥18G × 20 mm or larger

⑦ Other biopsy needle ( Fill in )

**(5) How many specimens do you sample for KB at a maximum?**

① 1 ② 2 ③ 3 ④ 4 ⑤ Not decided (depends on the case)

**(6) How many passes for sampling specimens are the upper limit for KB?**

① 1 ② 2 ③ 3 ④ 4 ⑤ 5 ⑥ 6 ⑦ 7 ⑧ 8 ⑨ 9 or more

⑩ Not decided (depends on the case) ⑪ Other option ( Fill in )

**(7) What maximal barrier precautions are included for KB? (Multiple selections allowed)**

① Cap ② Mask ③ Sterile body gown ④ Sterile gloves ⑤ Sterile drape ⑥ None of the above

**(8) Which of the following drugs or procedures are routinely used for KB? (Multiple selections allowed)**

① Antibiotics ② Atropine ③ Carbazochrome sodium sulfonate hydrate (Adona®)

④ Tranexamic acid ⑤ Indwelling urethral catheter ⑥ None of the above ⑦ Other ( Fill in )

**(9) Is there a target for blood pressure when KB is performed?** ① No ②Yes

**(10) What is the target systolic blood pressure (mmHg) for KB?**

① No target ② <160 ③ <150 ④ <140 ⑤ <130 ⑥ <120 ⑦ <110

**(11) What is the target dystolic blood pressure (mmHg) for KB?**

① No target ② <120 ③ <110 ④ <100 ⑤ <90 ⑥ <80 ⑦ <70 ⑧ <60

**(12) How do you administer antihypertensive drugs for patients with high blood pressure during KB?**

　 ① No drug use ② Oral administration ③ Sublingual administration

　 ③ Intravenous administration

**6. Sedation during pediatric kidney biopsy (KB)**

**※The questions below are only for pediatric nephrologists.**

**(1) What is the age indicated for intravenous (IV) anesthesia for pediatric KB in a hospital ward?**

　　① 3 yrs or younger ② 5 yrs or younger ③ 7 yrs or younger ④ 10 yrs or younger

⑤ 15 yrs or younger ⑥ All pediatric cases ⑦ Other ( Fill in )

**(2) Which of the following drugs are administered for IV anesthesia for pediatric KB?**

　　① Hydroxyzine ② Midazolam ③ Pentazocine ④ Ketamine ⑤ Thiopental or Thiamylal ⑥ Other drug or comment ( Fill in )

**(3) What is the age indicated for general anesthesia in the operating room for pediatric KB?**

　　① 3 yrs or younger ② 5 yrs or younger ③ 7 yrs or younger ④ 10 yrs or younger

⑤ 15 yrs or younger ⑥ All pediatric cases ⑦ Other ( Fill in )

**(4) What is the age indicated for open KB?**

　　① 3 yrs or younger ② 5 yrs or younger ③ 7 yrs or younger ④ 10 yrs or younger

⑤ 15 yrs or younger ⑥ All pediatric cases ⑦ Other ( Fill in )

**7. Hemostasis, bed rest, and examination after kidney biopsy (KB)**

**(1) Is manual compression on the biopsy site performed for hemostasis after KB?**

① No ② Yes, manual compression for ( Fill in ) minutes ③ Other ( Fill in )

**(2) Is a sandbag used for compression after KB?**

① No ② Yes, compression by a sandbag for ( Fill in ) hrs ③ Other ( Fill in )

**(3) Is abdominal taping or bandaging over the biopsy sites used for hemostasis after KB?**

① No ② Yes, abdominal taping or bandage for ( Fill in ) hrs ③ Other ( Fill in )

**(4) How long is strict bed rest in a supine position prescribed after KB?**

　 ① 0–2 hrs ② 2–4 hrs ③ 4–8 hrs ④ 8–16 hrs ⑤ 16–24 hrs ⑥ Over 24 hrs

⑦ Other ( Fill in )

**(5) How long is bed rest prescribed after KB?**

　 ① 0–2 hrs ② 2–4 hrs ③ 4–8 hrs ④ 8–16 hrs ⑤ 16–24 hrs ⑥ Over 24 hrs

⑦ Other ( Fill in )

**(6) How long is physical exercise avoided after KB?** ( Fill in ) days

**(7) When are discontinued anticoagulant or antiplatelet drugs restarted after KB?**

　　① No drug discontinuation before KB ② The day after KB ③ 2 days after KB ④ Other ( Fill in )

**(8) Is a hemostatic agent routinely used after KB?**

① No ② Yes (a) Carbazochrome sodium sulfonate hydrate (Adona®) (b) Tranexamic acid

(c) Other ( Fill in ) (multiple selections allowed)

**(9) When is a blood test routinely performed for monitoring after KB?**

① No test performed ② The day of KB ③ The day after KB ④ 2 days after KB

⑤ Other ( Fill in )

**(10) When is an ultrasound examination routinely performed to confirm hemostasis after KB?**

① No test performed ② Immediately after KB ③ 0–2 hrs ④ 2–4 hrs ⑤ 4–8 hrs

⑥ 8–16 hrs ⑦ 16–24 hrs ⑧ 24 hrs or later ⑨ Other ( Fill in )

**(11) Which of the following procedures will be adopted for hemostasis when severe bleeding occurs after KB?**

① Surgical procedure at my own hospital ② Surgical procedure at an affiliated hospital

③ Transcatheter arterial embolization (TAE) at my own hospital ④ TAE at an affiliated hospital

⑤ None of the above

**(12) Other comments about KB procedures in your facility** ( Fill in )

**8. Bleeding complications after kidney biopsy (KB) over the past 3 years**

| **Year** | **2015** | **2016** | **2017** |
| --- | --- | --- | --- |
| **The number of patients undergoing KB** | | | |
| **Total** |  |  |  |
| **Open KB** |  |  |  |
| **Laparoscopic KB** |  |  |  |
| **Number of patients with a bleeding complication after KB** | | | |
| **① Macroscopic hematuria with no treatment** |  |  |  |
| **② Bleeding complications with treatment** |  |  |  |
| **With blood transfusion** |  |  |  |
| **With surgical hemostasis** |  |  |  |
| **With TAE** |  |  |  |
| **With bladder lavage** |  |  |  |
| **With nephrectomy** |  |  |  |
| **Death from severe bleeding due to KB** |  |  |  |

**9. Fixation and processing of kidney biopsy (KB) specimens**

**(1) Which of the following KB specimens can you fix and process at your facility?**

**(Multiple selections allowed)**

① Light microscopy (LM) ② Immunofluorescence (IF) ③ Electron microscopy (EM)

**(2) Who divides biopsy specimens and puts them in the fixatives at your facility?**

① Biopsy operator (nephrologist) ② Assistant operator (nephrologist) ③ Nurse ④ Technician

⑤ Pathologist ⑥ Other ( Fill in )

**(3) Are KB specimens routinely assessed before division at your facility?**

① No ② Yes, using a dissecting microscope ③ Yes, using a light microscope

④ Other ( Fill in )

**(4) How do you keep KB specimens from drying before fixation at your facility?**

① We do nothing in particular ② We place specimens in normal saline

③ We wrap specimens with saline-soaked gauze ④ We place specimens in other liquid ( Fill in )

⑤ Other ( Fill in )

**(5) How are KB specimens divided for LM, IF, and EM at your facility?**

① Either end from all cores is taken for IF and EM, with the remainder divided for LM.

② Both ends from all cores are taken for IF and EM, with the remainder divided for LM.

③ Samples are randomly taken from all cores for IF, EM, and LM.

**(6) What is the formalin concentration used for fixation of KB specimen at your facility?**

① 10% ② 20% ③ Neutral buffered formalin ( Fill in )% ④ Other ( Fill in )

**(7) What fixatives are used for fixation of KB specimens at your facility?**

① Formalin only ② Other fixative only ( Fill in ) ③ Formalin and other fixative ( Fill in )

④ Other ( Fill in )

**(8) What is used for freezing specimens for processing at your facility?**

① Dry ice ② Dry ice with acetone ③ Hexane bottle in dry ice with acetone

④ Deep freezer at ( Fill in ) degrees centigrade ⑤ Liquid nitrogen ⑥ Other ( Fill in )
