# Supplementary material for: A nationwide survey on clinical practice patterns and bleeding complications of percutaneous native kidney biopsy in Japan
Source: Clin Exp Nephrol. 2020 Mar 18;24(5):389–401. doi: 10.1007/s10157-020-01869-w (PMC7174253; doi:10.1007/s10157-020-01869-w)
Supplement: Supplementary file 2 — Supplementary file2 (DOCX 27 kb) [file 10157_2020_1869_MOESM2_ESM.docx]

Supplement 2

**Questionnaire Form**

**Respondent’s specialty** ① Nephrology ② Pediatrics ③ Urology ④ Other ( Fill in )

**This is a questionnaire for the additional survey on transcatheter arterial embolization (TAE) as a severe bleeding complication of percutaneous native kidney biopsy (PNKB).**

**If you have had a patient in your facility who underwent TAE for critical hemorrhage after PNKB in the past 5 years, please answer the following questions.**

1. **Background of the patient with TAE after PNKB**

**(1) Please describe the clinical background at biopsy of the patient with TAE**

① Age ( ) years ② sex ( Male or Female ) ③ Height ( ) cm ④ Weight ( ) kg

⑤ Serum creatinine ( ) mg/dL ⑥ eGFR ( ) mL/min/1.73m^2^ ⑦ Hemoglobin ( ) g/dL

⑧ Platelet count ( ) × 10^4^/mm^3^ ⑨ Bleeding time ( ) minutes ⑩ PT-INR ( )

⑪ APTT ( ) seconds ⑫ Systolic/Diastolic blood pressure before PNKB ( / ) mmHg

⑬ Kidney size/Kidney major axis ( ) mm

**(2) Which of the following best describes the patient’s capacities to perform everyday activities?**

① Able to perform all physical activities of daily living (ADL), and reach places out of walking distance without assistance.

② Able to perform indoor physical ADL without assistance, but reaching places out of walking distance requires a walking stick or some help.

③ Lives out of a wheelchair and needs help regularly with certain physical activities.

④ Is bedridden and requires assistance throughout the day and/or night to carry out ADL.

**(3) Did the patient with TAE have diabetes mellitus?**

① No ② Yes

**(4) Was the patient with TAE at high risk of bleeding?**

① No ② Yes

**If yes, which of the following risk factors did the patient exhibit?**

① Unilateral kidney ② Hypoplastic kidney ③ Atrophic kidney ④ Cystic kidney disease

⑤ Malignant hypertension ⑥ Pregnancy ⑦ Other ( Fill in )

**(5) If the patient with TAE took an anticoagulant drug, was the anticoagulant still continued at biopsy?**

① No ② Yes

**(6) If the patient with TAE took an antiplatelet drug, was the antiplatelet still continued at biopsy?**

① No ② Yes

**(7) Which of the following was the patient’s clinical diagnosis at biopsy?**

① Chronic glomerulonephritis ② Rapidly progressive glomerulonephritis

③ Acute renal failure (e.g. drug-induced nephropathy) ④ Nephrotic syndrome

⑤ Monoclonal gammopathy ⑥ Anuria ⑦ Other ( Fill in )

**(8) Which of the following was the patient’s histological diagnosis by biopsy?**

① IgA nephropathy ② Crescentic glomerulonephritis ③ Diabetic nephropathy

④ Benign nephrosclerosis ⑤ Myeloma kidney/Amyloidosis ⑥ Other ( Fill in )

**2. Procedures of PNKB**

**(1) In what setting was the PNKB performed for the patient with TAE?**

① Inpatient setting ② Outpatient setting

**(2) How many PNKBs had the operators experienced before the biopsy for the patient with TAE?**

① 0-49 ② 50-199 ③ 200 or more ④ Other ( Fill in )

**(3) What size of biopsy needle for the PNKB was used in the patient with TAE?**

① 14 gauge (G) ② 16G ③ 17G ④ 18G ⑤ Other biopsy needle ( Fill in )

**(4) How many passes for sampling specimens were performed in the patient with TAE?**

① 1 ② 2 ③ 3 ④ 4 ⑤ 5 ⑥ 6 ⑦ 7 or more

**(5) How long was manual compression on the biopsy site performed for hemostasis after the PNKB for the patient with TAE?**

① 0–15 minutes ② 15–30 minutes ③ 30 minutes or longer ④Other ( Fill in )

**(6) How long was strict bed rest in a supine position after the PNKB for the patient with TAE?**

　 ① 0–2 hrs ② 2–4 hrs ③ 4–6 hrs ④ 6–8 hrs ⑤ 8-12 hrs ⑥ 12-18 hrs ⑦ Other ( Fill in )

**(7) How long was total bed rest after PNKB for the patient with TAE?**

　 ① 0–8 hours (hrs) ② 8–16 hrs ③ 16–24 hrs ④ Over 24 hrs ⑤ Originally bed-bound

⑥ Other ( Fill in )

**(8) Was a hemostatic agent used after the PNKB for the patient with TAE?**

① No ② Yes

**3. The timing and type of bleeding requiring TAE after the PNKB**

**(1) When did the severe bleeding requiring TAE become apparent after PNKB?**

① <6 hours ② <12 hours ③ <24 hours ④<7 days ⑤ ≥7 days

**(1) What type of bleeding requiring TAE occurred after PNKB?**

① Peri-nephritic (PN) or intra-nephritic (IN) bleeding ② Macrohematuria without PN or IN bleeding

③ Other ( Fill in )
